# Non-beta-amyloid/tau cerebrospinal fluid markers inform staging and progression in Alzheimer’s disease

**DOI:** 10.1186/s13195-018-0426-3

**Published:** 2018-09-25

**Authors:** Umesh Gangishetti, J. Christina Howell, Richard J. Perrin, Natalia Louneva, Kelly D. Watts, Alexander Kollhoff, Murray Grossman, David A. Wolk, Leslie M. Shaw, John C. Morris, John Q. Trojanowski, Anne M. Fagan, Steven E. Arnold, William T. Hu

**Affiliations:** 1 Department of Neurology, Emory University, 615 Michael Street, 505F, Atlanta, GA 30322 USA; 2 Department of Alzheimer’s Disease Research Center, Emory University, Atlanta, GA USA; 3 Knight Alzheimer’s Disease Research Center, Washington University, St. Louis, MO USA; 4 Department of Pathology, Washington University, St. Louis, MO USA; 5 Department of Neurology, Washington University, St. Louis, MO USA; 6 Penn Memory Center, University of Pennsylvania, Philadelphia, PA USA; 7 Center for Neurodegenerative Disease Research, University of Pennsylvania, Philadelphia, PA USA; 8 Penn FTD Center, University of Pennsylvania, Philadelphia, PA USA; 9 Department of Neurology, University of Pennsylvania, Philadelphia, PA USA; 10 Department of Pathology and Laboratory Medicine, University of Pennsylvania, Philadelphia, PA USA; 11 Present Address: Massachusetts General Hospital, Boston, MA USA

**Keywords:** Biomarkers, Fatty acid binding protein, Interleukin-10, Mild cognitive impairment, Neurofilament light chain

## Abstract

**Background:**

Alzheimer’s disease (AD) is a complex neurodegenerative disorder characterized by neuropathologic changes involving beta-amyloid (Aβ), tau, neuronal loss, and other associated biological events. While levels of cerebrospinal fluid (CSF) Aβ and tau peptides have enhanced the antemortem detection of AD-specific changes, these two markers poorly reflect the severity of cognitive and functional deficits in people with altered Aβ and tau levels. While multiple previous studies identified non-Aβ, non-tau proteins as candidate neurodegenerative markers to inform the A/T/N biomarker scheme of AD, few have advanced beyond association with clinical AD diagnosis. Here we analyzed nine promising neurodegenerative markers in a three-centered cohort using independent assays to identify candidates most likely to complement Aβ and tau in the A/T/N framework.

**Methods:**

CSF samples from 125 subjects recruited at the three centers were exchanged such that each of the nine previously identified biomarkers can be measured at one of the three centers. Subjects were classified according to cognitive status and CSF AD biomarker profiles as having normal cognition and normal CSF (*n* = 31), normal cognition and CSF consistent with AD (*n* = 13), mild cognitive impairment and normal CSF (*n* = 13), mild cognitive impairment with CSF consistent with AD (*n* = 23), AD dementia (*n* = 32; CSF consistent with AD), and other non-AD dementia (*n* = 13; CSF not consistent with AD).

**Results:**

Three biomarkers were identified to differ among the AD stages, including neurofilament light chain (NfL; *p* < 0.001), fatty acid binding protein 3 (Fabp3; *p* < 0.001), and interleukin (IL)-10 (*p* = 0.033). Increased NfL levels were most strongly associated with the dementia stage of AD, but increased Fabp3 levels were more sensitive to milder AD stages and correlated with both CSF tau markers. IL-10 levels did not correlate with tau biomarkers, but were associated with rates of longitudinal cognitive decline in mild cognitive impairment due to AD (*p* = 0.006). Prefreezing centrifugation did not influence measured CSF biomarker levels.

**Conclusion:**

CSF proteins associated with AD clinical stages and progression can complement Aβ and tau markers to inform neurodegeneration. A validated panel inclusive of multiple biomarker features (etiology, stage, progression) can improve AD phenotyping along the A/T/N framework.

## Background

The clinicopathologic description of Alzheimer’s disease (AD) underwent recent revisions to better characterize, on parallel continuums, the cognitive and neuropathologic features associated with beta-amyloid (Aβ) deposition, tau hyperphosphorylation, and neurodegeneration [1–5]. This A/T/N framework has the advantage of providing a multidimensional view of AD, although accurate antemortem detection of all three features remains an obstacle in early diagnosis and clinical trial design. AD biomarkers, including cerebrospinal fluid (CSF) [6] or positron emission tomography (PET) [7, 8] measures of amyloid and tau proteins, correlate well with postmortem amyloid and tau (A/T) pathology, but their levels have not been shown to accurately track disease progression [9, 10] to provide information on neurodegeneration. We and others have previously identified CSF proteins which accompany altered amyloid and tau biomarkers in large discovery cohorts, and these non-Aβ, non-tau (NANT) markers are candidate markers of neurodegeneration [11–17]. However, successful replication of these markers' association with AD has been challenging. This may be due to many issues, including recruitment bias [18], processing artifacts when assays are performed by commercial vendors [19], and different antibodies, and few of them have been replicated across cohorts and assay platforms to undergo further standardization and application.

CSF is a ready source for simultaneously testing multiple markers reflecting AD core pathology, copathology (ischemia, Lewy bodies), neurodegeneration, common biological alterations (e.g., neuroinflammation), and unique exposures (e.g., environmental toxins) [20]. We previously sought to identify NANT biomarkers through single-center studies [12, 14, 19], and subsequently determined that some replication failures resulted from biases in recruitment, diagnosis, preanalytical handling, and analytical algorithms [18]. To validate the association between AD pathology, neurodegeneration, and the top NANT biomarkers, we adapted a round-robin design [21] involving subjects recruited from three Alzheimer’s disease centers, and collaboratively measured levels of nine analytes to correlate with AD biomarkers and clinical AD stages.

## Methods

### Standard protocol approvals, registrations, and patient consents

The protocols were approved by the Institutional Review Boards (IRB) at Emory University (Emory), University of Pennsylvania (Penn), and Washington University (WU). Banked CSF samples were used for this study, and all subjects had previously consented to the long-term storage and subsequent analysis of CSF samples. Frozen CSF samples were exchanged among the centers under six bilateral material transfer agreements.

### Subjects and preanalytical processing

Demographic (age, sex, education), diagnostic (syndrome, global Clinical Dementia Rating (CDR), Mini-Mental State Examination (MMSE)), and *APOE* allelic information were collected by each center (Table 1). At Emory, CSF was collected via syringe between 08.00 and 12.00 without overnight fasting using 24-G Sprotte needles, placed in polypropylene tubes, and immediately aliquoted without centrifugation, labeled, and frozen at −80 °C. At Penn, CSF was collected by gravity or syringe without overnight fasting in the morning using 24-G Sprotte needles, placed in polypropylene tubes, transferred locally, aliquoted without centrifugation, labeled, and frozen at −80 °C. At WU, CSF was collected at 08.00 following overnight fasting via gravity using 22-G Sprotte needles, placed in polypropylene tubes, centrifuged at low speed to pellet any cellular debris, aliquoted, and frozen at −80 °C. All samples were shipped to the two external sites overnight on dry ice and immediately placed at −80 °C until analysis.

**Table 1:** Demographic features of subjects included in the current study

	NC^–^(*n* = 31)	NC^+^(*n* = 13)	MCI^–^(*n* = 13)	MCI^+^(*n* = 23)	AD dementia(*n* = 32)	OD(*n* = 13)
Female, *n* (%)	19 (61%)	9 (69%)	7 (54%)	13 (56%)	21 (66%)	5 (39%)
Age (years)	69.1 ± 6.3	74.6 ± 6.9	70.1 ± 5.0	70.6 ± 6.2	72.8 ± 7.1	65.4 ± 4.5
Caucasian, *n* (%)	28 (90%)	13 (100%)	12 (92%)	23 (100%)	30 (94%)	13 (100%)
Has at least one *APOE* ε4 allele, %	26%	38%	3/10 (30%)	52%	72%	31%
Education (years)	15.7 ± 3.3	15.4 ± 3.2	15.5 ± 3.2	15.0 ± 2.8	14.7 ± 3.8	15.4 ± 1.7
MMSE	28.9 ± 1.8	28.5 ± 1.8	27.4 ± 2.1	26.4 ± 2.5	21.7 ± 4.9	23.2 ± 6.3
Recruiting center, *n*						
Emory	9	3	7	7	10	8
Penn	12	0	6	6	12	5
WU	10	10	0	10	10	0
Emory AD biomarkers (Luminex)						
Aβ42 (pg/mL)	301.3 ± 106.6	189.1 ± 134.8	306.3 ± 98.5	184.1 ± 68.6	171.3 ± 37.6	232.2 ± 114.9
t-Tau (pg/mL)	48.5 ± 22.4	77.3 ± 53.3	63.8 ± 27.9	150.4 ± 81.6	148.8 ± 52.3	90.8 ± 106.0
p-Tau_181_ (pg/mL)	28.0 ± 10.1	53.4 ± 26.9	32.5 ± 6.9	63.3 ± 19.3	74.7 ± 16.6	27.6 ± 15.7
Penn AD biomarkers (Luminex)						
Aβ42 (pg/mL)	262.6 ± 72.5	N/A	242.1 ± 53.1	119.6 ± 18.0	121.9 ± 35.0	310.6 ± 72.9
t-Tau (pg/mL)	54.4 ± 13.1	N/A	76.9 ± 34.8	96.6 ± 64.9	117.9 ± 41.4	66.2 ± 28.3
p-Tau_181_ (pg/mL)	19.3 ± 14.4	N/A	33.9 ± 34.2	36.6 ± 21.1	45.6 ± 22.6	15.2 ± 4.5
WU AD biomarkers (ELISA)						
Aβ42 (pg/mL)	736.9 ± 152.7	369.2 ± 87.5	N/A	349.0 ± 121.8	291.7 ± 74.4	N/A
t-Tau (pg/mL)	282.8 ± 115.7	359.5 ± 230.3	N/A	615.2 ± 177.7	628.2 ± 363.1	N/A
p-Tau_181_ (pg/mL)	54.4 ± 19.1	74.7 ± 40.7	N/A	107.4 ± 52.3	90.7 ± 52.7	N/A

Values are shown as mean ± standard deviation unless otherwise indicated

*Aβ* beta-amyloid, *AD* Alzheimer’s disease, *ELISA* enzyme-linked immunosorbent assay, *Emory* Emory University, *MCI* mild cognitive impairment, *MMSE* Mini-Mental State Examination, *N/A* not available, *NC* normal cognition, *OD* other non-AD dementia, *Penn* University of Pennsylvania, *p-Tau*_*181*_ phosphorylated tau, *t-Tau* total tau, *WU* Washington University

### Subject grouping

Each subject was categorized according to clinical diagnosis (normal cognition (NC), mild cognitive impairment/very mild dementia/CDR 0.5 (MCI), AD dementia, and other non-AD dementia (OD)), and those with NC or MCI were further stratified according to CSF AD biomarkers. In all subjects, CSF levels of Aβ42, total tau (t-Tau), and tau phosphorylated at threonine 181 (p-Tau_181_) had been previously measured using INNO-BIA Alzbio3 (Emory [6], Penn [22]) or INNOTEST® (WU) [23] following the manufacturer’s protocols (Fujirebio US, Malvern, PA). All three centers included subjects with NC without CSF biomarkers consistent with AD (NC^–^), MCI with CSF consistent with AD (MCI^+^), and AD dementia. In addition, Emory and WU included NC subjects with CSF biomarkers consistent with AD (NC^+^), and Emory and Penn included MCI subjects with CSF not consistent with AD (MCI^–^) as well as subjects with OD (Table 1). The diagnosis for OD includes behavioral variant frontotemporal dementia (*n* = 5), semantic variant of primary progressive aphasia (*n* = 1), progressive supranuclear palsy (*n* = 2), and dementia with Lewy bodies (*n* = 5).

### NANT biomarker assays

Nine NANT analytes were selected by WTH, AMF, and SEA for validation based on previous biomarker discovery studies, and assay development and performance took place at Emory (interleukin (IL)-7, IL-10, fractalkine, tumor necrosis factor (TNF)-α), Penn (fatty acid binding protein 3 (Fabp3), insulin-like growth factor binding protein 2 (IGF-BP2), neurofilament light chain (NfL)), and WU (monocyte chemotactic protein 1 (MCP1), chitinase-3-like protein 1 (YKL-40)). At Emory, IL-7, IL-10, fractalkine, and TNF-α levels (Milliplex MAP Human Cytokine Panel, HCYTOMAG-60 K, EMD Millipore, Billerica, MA) were measured in a Luminex 200 platform following the manufacturer’s protocol except that two 100-μL aliquots of CSF were used for duplicates. At Penn, plate-based enzyme-linked immunosorbent assays (ELISAs) were performed according to the manufacturer’s instructions for human IGFBP-2 (Sigma, St. Louis, MO; cat. no. RAB0233), human FABP3 (EMD Millipore; cat. no. EZFABP3-38 K), and human neurofilament-light RUO (IBL International, Hamburg, Germany; cat. no. UD51001). At WU, MCP1 levels were analyzed in a Luminex 200 platform (Milliplex MAP Human Adipocyte Panel, HADCYMAG-61 K; EMD Millipore, Billerica, MA), and YKL-40 levels were measured using ELISA (MicroVue YKL-40 EIA Kit, Quidel, San Diego, CA) [11]. All operators were blinded to the diagnosis, and final assay results were collected at Emory for analysis.

### Effects of centrifugation

Because CSF samples were centrifuged after collection at WU but not centrifuged at Emory and Penn, we performed prospective experiments at Emory to determine the effect of prefreezing centrifugation. Specifically, after CSF was collected from 16 subjects, CSF samples were immediately divided into two equal portions. One portion was centrifuged at 2000 g and 4 °C for 10 min while the other portion was kept on ice. The supernatant from the centrifuged portion was carefully aliquoted, labeled, and frozen at –80 °C until analysis, and the noncentrifuged portion was similarly aliquoted, labeled, and frozen at –80 °C until analysis. Levels of two analytes whose levels varied according to center (IL-7, IL-10) and one analyte whose level did not vary according to center (NfL) were analyzed in samples with and without centrifugation.

### Statistical analysis

Statistical analysis was performed by IBM-SPSS 24 (Chicago, IL) at Emory. For baseline comparison among the three centers, Chi-squared tests for categorical variables and analysis of variance (ANOVA) for continuous variables were used to determine differences. *APOE* genotyping was not available for 3 MCI^–^ subjects from Emory. Since MCI^–^ and OD were included for comparative purposes, these missing genotypes did not influence the study’s main analysis.

For biomarker levels, ANOVA showed that three analytes (IL-7, IL-10, and MCP-1) differed significantly among the recruiting centers. To standardize data handling and to account for these center-associated differences, a site-specific *Z* score was created for each analyte using the mean and standard deviation of the combined NC^–^ and AD dementia cohort. After *Z* transformation, the levels of each analyte were confirmed to be normally distributed by Kolmogorov-Smirnov tests. Student’s *t* tests were then performed to identify analytes whose levels differed between NC^–^ and AD dementia, with a false discovery rate (FDR) threshold of 0.10 to account for multiple comparisons. Student’s *t* tests were also used to determine whether prefreezing centrifugation affected biomarker levels.

Analysis of covariance (ANCOVA) was used to determine biomarkers that can differentiate among the four theoretical stages of AD development (NC^–^, NC^+^, MCI^+^, AD dementia), adjusting for age, sex, *APOE* ε4 status, and recruiting center. A threshold of 0.10 for FDR was selected to account for multiple comparisons. Pearson’s correlation was then used to analyze the relationships between established CSF AD biomarkers (Aβ42, t-Tau, p-Tau_181_) and the three biomarkers identified through ANCOVA.

Finally, for correlation between baseline IL-10 levels and rates of longitudinal cognitive decline, mixed linear modeling was used to determine whether IL-10 levels were associated with faster rates of cognitive decline. *Z* scores for executive, memory, language, and visual spatial domains were calculated as previously described. In the mixed linear model, domain-specific *Z* scores were entered as the dependent variable; gender, race, t-Tau (previously found to influence rates of cognitive decline) [24], IL-10, time, time × IL-10, age, and education were entered as fixed variables, and time was also entered as a random variable. IL-10 was considered to significantly influence the rates of longitudinal decline if the interaction term time × IL-10 was associated with domain-specific *Z* scores at *p* < 0.01 to adjust for multiple comparisons.

## Results

The overall cohort included 125 subjects, including 31 NC^–^, 13 NC^+^, 12 MCI^–^, 24 MCI^+^, 32 AD dementia, and 13 OD. Subjects were younger (68.7 vs. 73.4 years, *p* = 0.003) and more educated (15.7 vs. 14.0 years, *p* = 0.025) at Emory than WU. Neither site differed from Penn. All three sites were otherwise similar for sex (*p* = 0.564), race (*p* = 0.418), and *APOE* ε4 status (*p* = 0.445).

### NANT biomarkers associated with AD dementia

**Fig. 1:**
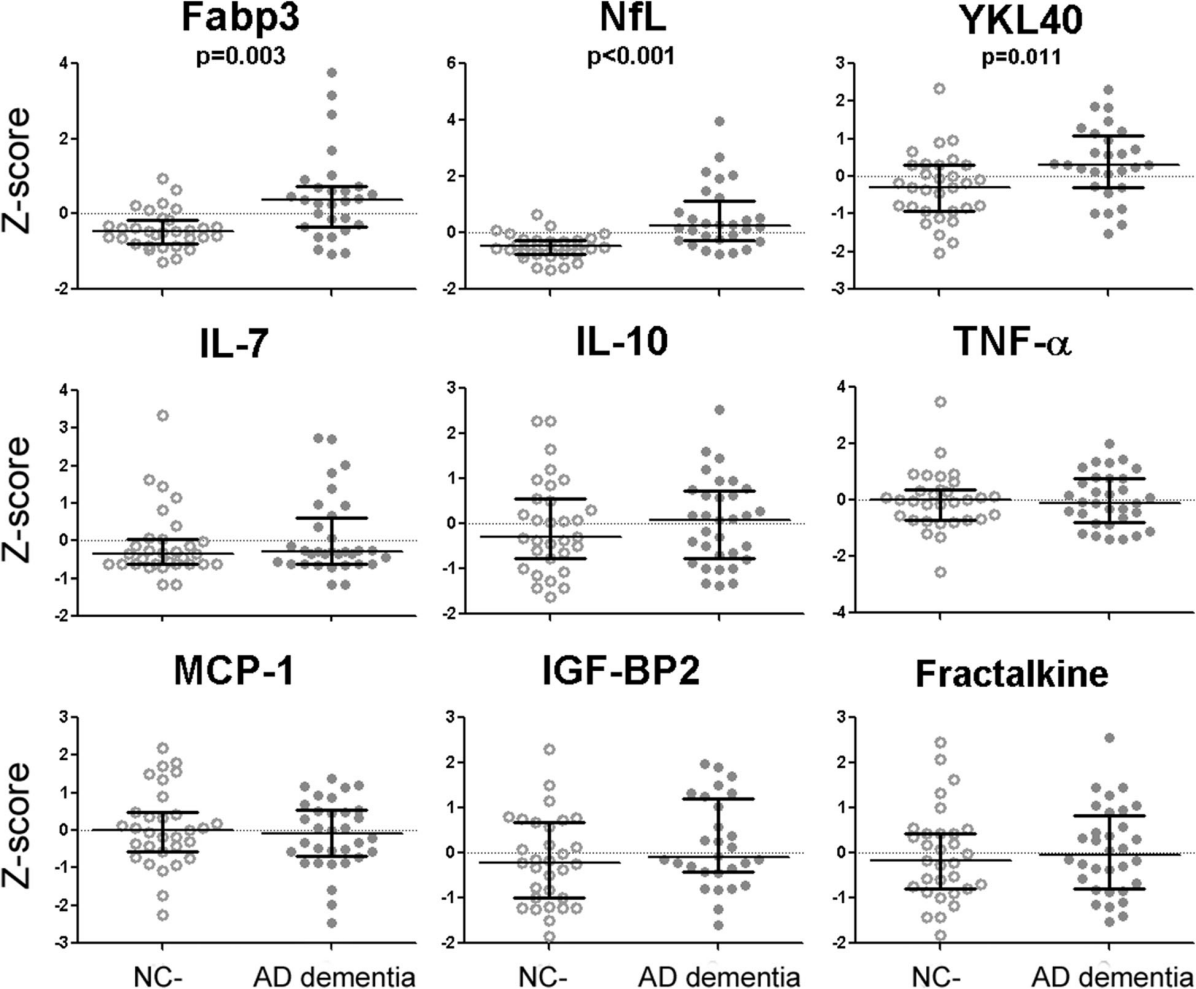
CSF analyte levels (*Z* scores) for the combined cohort of normal cognition without CSF biomarkers consistent with Alzheimer’s disease (NC^–^) and Alzheimer’s disease (AD) dementia subjects. To account for inter-center variability, a center-specific *Z* score was calculated for each analyte by grouping NC^–^ and AD dementia subjects together to calculate the group mean and standard deviation. Student’s *t* tests were then used to compare the *Z* scores of NC^–^ and AD dementia subjects across the three centers, with FDR < 5%. Bars represent median and interquartile ranges, and the unadjusted *p* values are shown. Fabp3 fatty acid binding protein 3, IL interleukin, MCP-1 monocyte chemotactic protein 1, NfL neurofilament light chain, TNF tumor necrosis factor, YKL40 chitinase-3-like protein 1

Since prior NANT biomarker studies sought biomarkers that distinguished between subjects with NC (NC^–^ with or without NC^+^) and AD dementia, we first analyzed whether levels of the nine candidate biomarkers differed between NC^–^ and AD dementia. This identified three analytes (NfL, Fabp3, and YKL-40) associated with AD dementia after adjusting for FDR of 5% (Fig. 1). None of the other analytes differed between NC^–^ and AD dementia (adjusted *p* value range of 0.252 to 0.977). Controlling for age, sex, center of recruitment, and *APOE* ε4 status slightly diminished the significance of YKL-40 (*p* = 0.062) but showed similar results for NfL (*p* < 0.001) and Fabp3 (*p* < 0.001). Thus, NfL and Fabp3 best distinguished between the two extreme categories (NC^–^ and AD dementia).

### NANT biomarkers associated with AD stages

**Fig. 2:**
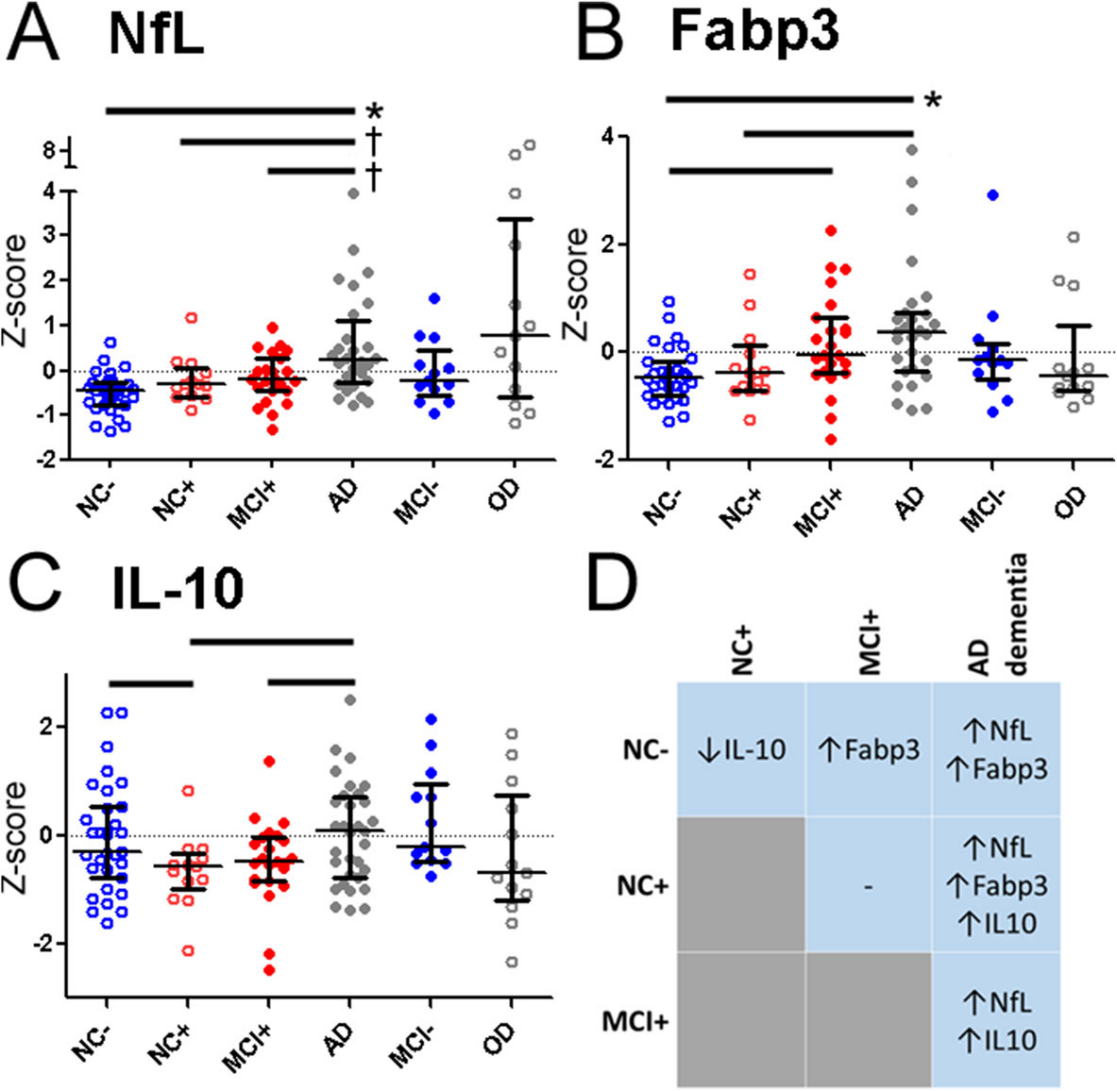
CSF levels (*Z* scores) of neurofilament light chain (NfL) (**a**), fatty acid binding protein 3 (Fabp3) (**b**), and interleukin (IL)-10 (**c**) in subjects with normal cognition (NC), mild cognitive impairment (MCI), Alzheimer’s disease (AD) dementia, and other non-AD dementia (OD). (*p < 0.001; ^†^*p* < 0.005; *p* < 0.05 for other comparisons indicated). Differences between different subgroups are summarized in **d**, with direction of change reflecting the stage with more severe pathology or cognitive impairment

Since the clinical manifestation of AD neuropathology is hypothesized to progress through the presymptomatic, MCI, and dementia stages, we next examined in this cross-sectional cohort whether levels of the candidate analytes differed among NC^–^, NC^+^, MCI^+^, and AD dementia through ANCOVA adjusting for age, sex, education, and presence of *APOE* ε4 allele. This confirmed NfL (F(3,94) = 9.455, *p* < 0.001) and Fabp3 (F(3,94) = 5.869, *p* < 0.001) to be associated with AD stages. Specifically, NfL levels were higher in AD dementia than NC^–^, NC^+^, or MCI^+^ (Fig. 2a), and Fabp3 levels were higher in AD dementia than NC^–^ and NC^+^, and higher in MCI^+^ than NC^–^ (Fig. 2b). Furthermore, IL-10 (F(3,94) = 3.034, *p* = 0.033) showed stage-associated differences, with NC^+^ having lower IL-10 levels than NC^–^, but AD dementia having higher IL-10 levels than NC^+^ or MCI^+^ (Fig. 2c). No biomarkers significantly differed in their level between NC^+^ and MCI^+^ (Fig. 2d).

### NfL and Fabp3 levels associated with CSF tau biomarkers

**Fig. 3:**
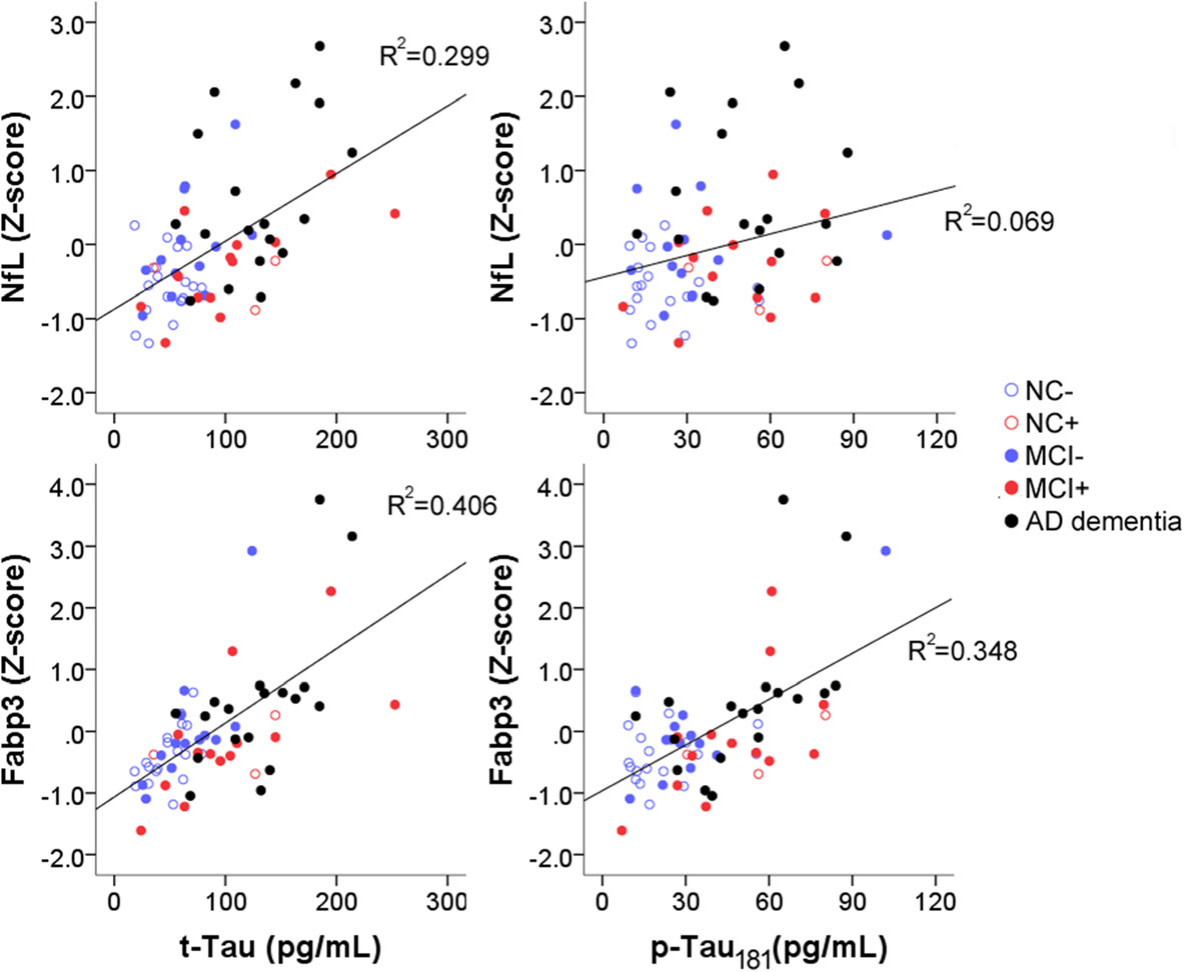
Correlations between CSF tau-related proteins and neurofilament light chain (NfL) and fatty acid binding protein 3 (Fabp3) levels. Fabp3 levels correlated strongly with total tau (t-Tau) and phosphorylated tau (p-Tau_181_) levels, while NfL levels correlated better with t-Tau than *p*-Tau_181_ levels. AD Alzheimer’s disease, MCI mild cognitive impairment, NC normal cognition

As emerging AD therapeutics often target amyloid and tau, levels of established CSF AD biomarkers (Aβ42, t-Tau, and p-Tau_181_) may serve better to inform target engagement than treatment-associated downstream effects. We therefore analyzed if CSF NfL, Fabp3, and IL-10 correlated with the established CSF AD biomarkers (Aβ42, t-Tau, and p-Tau_181_) to serve as downstream markers. Analyzing samples from Emory and Penn (where established biomarker assays had been performed on identical Luminex platforms) together, both CSF NfL and Fabp3 levels correlated strongly with CSF t-Tau levels (*p* < 0.001), and CSF Fabp3 (*R*^2^ = 0.348, *p* < 0.001) levels better correlated with CSF p-Tau_181_ levels than CSF NfL levels (*R*^2^ = 0.069, *p* = 0.035, not significant after correction for multiple comparisons; Fig. 3). A similar trend was seen in samples from WU (where established biomarker assays were performed by ELISA), with Fabp3 levels correlating with t-Tau (*p* < 0.001) and p-Tau (*p* < 0.001), and NfL levels correlating better with t-Tau (*p* < 0.001) than p-Tau_181_ (*p* = 0.074). None of the NANT biomarkers correlated with CSF Aβ42, and diagnosis did not influence the relationship between tau biomarkers and the two novel biomarkers (Fabp3 and NfL).

### IL-10 associated with rates of longitudinal cognitive decline in MCI^+^

**Fig. 4:**
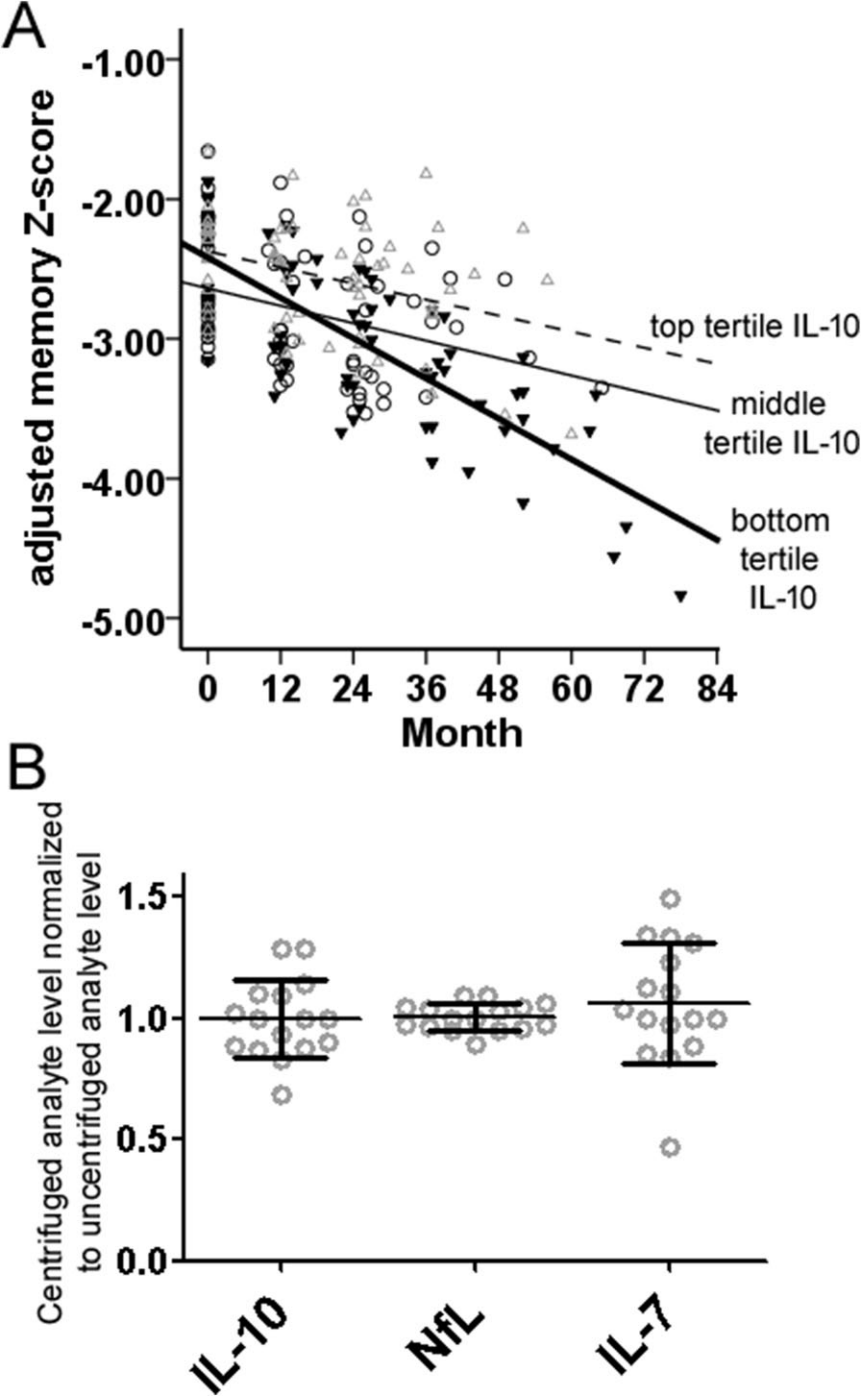
Relationship between CSF interleukin (IL)-10 levels, rates of cognitive decline, and preanalytical processing. Lower CSF IL-10 levels were associated with greater decline in memory functions (adjusting for age, gender, race, education) in MCI^+^. **a** Memory *Z* scores were derived from averaging verbal and visual delayed recall *Z* scores. Mixed linear modeling was performed using IL-10 as a continuous variable (*p* = 0.005), and IL-10 levels are shown as tertiles for illustrative purposes (open triangle, open circle, and filled triangle represent top, middle, and bottom quartiles). **b** Centrifugation of CSF after collection but before freezing did not alter IL-10 levels or levels of two other biomarkers (neurofilament light chain (NfL) and IL-7)

Because CSF IL-10 levels did not correlate with t-Tau or p-Tau_181_, we then analyzed if CSF IL-10 levels correlated with rates of decline in MCI^+^ subjects since clinicians often consider longitudinal decline as an important feature of MCI^+^. This may introduce bias into the selection of MCI^+^ subjects, especially when IL-10 levels did not differ between NC^–^ and AD. Mixed linear modeling showed that, in a group of 51 MCI^+^ subjects longitudinally followed at Emory (median follow-up 36 months, range 18–78 months), lower IL-10 levels were associated with greater rates of decline in memory *Z* scores (*p* = 0.006 for time × IL-10 levels; Table 2 and Fig. 4a), but not in executive (*p* = 0.270), language (*p* = 0.246), or visual spatial (*p* = 0.975) *Z* scores. In comparison, higher CSF t-Tau levels were associated with worse memory *Z* scores, but neither CSF t-Tau nor p-Tau_181_ influenced the rates of memory decline.

**Table 2:** Mixed linear model analysis of memory *Z* scores in MCI^+^ subjects longitudinally characterized at Emory (*n* = 51)

	Coefficient (95% confidence interval)	*p*
Age	0.020 (−0.011, 0.051)	0.210
Male gender	0.613 (0.148, 1.078)	0.011
Minority race	0.210 (−0.647, 1.067)	0.625
Education (years)	0.029 (−0.057, 0.116)	0.505
t-Tau (pg/mL)	−0.002 (−0.005, −0.001)	0.073
IL-10 level (pg/mL)	−0.035 (−0.139, 0.069)	0.510
Time (months)	−0.043 (−0.060, −0.026)	< 0.001
Time × IL-10 (months × pg/mL)	0.003 (0.001, 0.005)	0.006

*IL* interleukin, *MCI* mild cognitive impairment, *t-Tau* total tau

### NANT biomarker levels not associated with prefreezing CSF centrifugation

Finally, we sought to determine whether prefreezing CSF centrifugation (performed at WU) represented another bias in measured NANT levels since centrifuged samples represented 77% of NC^+^ and 43% of MCI^+^ cases. Centrifuged and noncentrifuged samples prospectively collected from the same individuals at Emory showed similar absolute levels of IL-10 and NfL (Fig. 4b), suggesting that their association with AD stages was independent of the preanalytical processing differences between the centers. In keeping with this, levels of IL-7 (which showed a large inter-site difference) were also not influenced by centrifugation.

## Discussion

Reproducible NANT biomarkers associated with AD pathogenesis or progression have the potential for complementing existing cognitive/functional assessments and improving clinical trial designs. Here we used multicentered samples and independent assays to confirm CSF Fabp3 and NfL as stage-dependent biomarkers in AD. The levels of these two markers also correlated with t-Tau (both) and p-Tau_181_ (Fabp3) in the CSF, and can be prospectively tested as surrogate markers of response in future clinical trials targeting tau. Furthermore, we found a complex relationship between CSF IL-10 levels, AD, and cognition, but associated lower CSF IL-10 levels to faster cognitive decline in MCI. Altogether, these findings point to a set of unique biochemical events associated with cumulative and on-going cognitive decline in AD, and add a suite of NANT biomarkers to the A/T/N scheme.

Previous work—including our own—has primarily focused on NANT biomarkers whose levels differed between NC^–^ and AD dementia. Subjects with normal cognition but abnormal AD biomarkers (CSF or PET) were variably included with or excluded from those whose cognition and AD biomarkers were both normal, and the distinction between MCI^+^ and AD dementia could be based on the number of impaired neuropsychological domains, functional independence, or consensus. Aside from these study design biases, our current study showed that analyzing only the two extreme groups overlooked at least one biologically meaningful marker, IL-10. At the same time, levels of the most commonly cited candidate staging marker—NfL, a neuronal cytoskeletal protein associated with axonal injury—were most elevated in the dementia stage of AD, but did not sufficiently distinguish between the earlier stages (NC^–^, NC^+^, MCI^+^) nor correlate strongly with p-Tau_181_. The difference in NfL observed here is in line with findings from the Alzheimer’s Disease Neuroimaging Initiative (ADNI) and favors NfL more as a marker of staging/progression in neurodegenerative disorders with faster progression (e.g., frontotemporal dementia) than typical AD. Similarly, the difference in YKL-40 levels (a glycoprotein secreted by astrocytes and infiltrating macrophages) was consistent with previously reported ranges [11].

Fabp3 levels better distinguished between different AD stages than NfL and YKL-40 [11], and may serve as a good neurodegenerative biomarker since its levels correlated well with CSF t-Tau and p-Tau_181_ levels. Fabp3 is a small soluble protein expressed in neurons, astrocytes, and brain endothelial cells [25–27]. It is involved in the intracellular transport of polyunsaturated fatty acid [28] as well as modulation of acetylcholine and glutamate release [29]. Brains with AD and schizophrenia were found to have reduced Fabp3 levels [30, 31], and serum Fabp3 levels are elevated in multiple dementia and brain injury syndromes [32–34]. Data from the ADNI and other studies have shown that CSF Fabp3 levels do not differ between NC^–^ and NC^+^ [35, 36], but do increase in the symptomatic AD stages [36, 37] and with progressive entorhinal atrophy [38]. Consistent with these prior findings, we also found similar Fabp3 levels in MCI^+^ and AD dementia. Thus, whereas increased NfL levels may reflect sufficient neurodegeneration to result in functional decline [39], Fabp3 may be a more sensitive marker to predementia neurodegeneration.

We found IL-10 levels to differ between clinical AD stages but not between NC^–^ and AD dementia. This came as counter-intuitive for us, which led to further experiments related to IL-10. CSF IL-10 levels were variably linked with AD in previous discovery-based studies [12, 14]. Among potential explanations for these discrepant findings, we eliminated analytical and preanalytical variabilities as confounds in our study since IL-10 levels were all measured at a single site and did not differ in a prospective follow-up study targeting the effects of pre-freezing centrifugation. At the same time, selection bias in banked biospecimens may account for reduced IL-10 levels in MCI^+^ compared with AD as lower IL-10 levels were associated with greater rates of memory decline, a feature often considered when MCI samples are selected retrospectively. This is supported by our follow-up study where MCI subjects with the lowest CSF IL-10 levels tended to experience greater memory decline. At the same time, there exist potential biological explanations for lower IL-10 levels in NC^+^ and MCI^+^. IL-10 has often been considered an anti-inflammatory cytokine, but it is released by proinflammatory, anti-inflammatory, and regulatory T helper cells. Its release and effects are thus complex, and IL-10 does not exist or act in isolation. Lower IL-10 levels in NC^+^ may be interpreted as a failure in anti-inflammatory processes associated with onset of pathologic AD, or alternatively balanced anti- and proinflammatory responses in asymptomatic AD (e.g., we previously showed complement activation to accompany the MCI^+^ to AD transition [18]). Similarly, higher IL-10 levels in AD than NC^+^ and MCI^+^ may represent exaggerated anti-inflammatory responses or appropriate IL-10 response to AD-related neuroinflammation. These challenges call for the simultaneous measurements of cytokines representing different pro- and anti-inflammatory pathways in future studies, as well as immunophenotyping analysis in the CSF. This approach will also better explain why reduced IL-10 levels may predict faster rates of decline in MCI.

Instead of measuring promising AD biomarkers only at a single site (academic or commercial), we show here that a collaborative model of replication moves the most promising NANT biomarkers towards further development. It enables a greater number of candidate markers to undergo simultaneous validation in subjects recruited from each center in a head-to-head design, identifies analytes with inter-site variabilities, and permits follow-up experiments to empirically determine the effects of different preanalytical procedures. At the same time, our study is limited by the sample size, as yet unidentified factors to account for center-to-center variations, genetic background of populations at the three geographically separate sites, and imperfect matching of some diagnostic categories among centers (NC^+^, OD). We did not include CSF biomarkers for non-beta-amyloid/tau neurodegenerative processes (e.g., a-synuclein, phosphorylated TDP-43 levels) as they are less mature, and accounting for them may help explain variability across centers and AD stages. We also did not analyze the NANT biomarker levels in a large group of OD since cases with high confidence pathology (through autopsy confirmation or, less preferably, mutation because of the mutations’ potential direct impact on inflammation) are limited in number. Translation of promising markers validated here into the A/T/N biomarker suite will need to prospectively determine the impact of biological, preanalytical, and analytical variabilities on the levels and stability of these markers, and the A/T/N scheme itself may need future revision to account for copathology and other contributors.

## Conclusion

In summary, we successfully confirmed three proteins (Fabp3, NfL, and IL-10) as potentially informative biomarkers to complement established AD biomarkers (Aβ and tau) through a three-centered, North American, non-ADNI study. Importantly, we used assays easily accessible to investigators who can further optimize their development and translation.

## Abbreviations

Aβ: Beta-amyloid
AD: Alzheimer’s disease
ADNI: Alzheimer’s Disease Neuroimaging Initiative
ANCOVA: Analysis of covariance
ANOVA: Analysis of variance
CDR: Clinical Dementia Rating
CSF: Cerebrospinal fluid
ELISA: Enzyme-linked immunosorbent assay
Emory: Emory University
Fabp3: Fatty acid binding protein 3
FDR: False discovery rate
IGF-BP2: Insulin-like growth factor binding protein 2
IL: Interleukin
MCI: Mild cognitive impairment
MCP1: Monocyte chemotactic protein 1
MMSE: Mini-Mental State Examination
NANT: Non-beta-amyloid, non-tau
NC: Normal cognition
NfL: Neurofilament light chain
OD: Other non-Alzheimer’s disease dementia
Penn: University of Pennsylvania
PET: Positron emission tomography
p-Tau_181_: Tau phosphorylated at threonine 181
TNF: Tumor necrosis factor
t-Tau: Total tau
WU: Washington University
YKL-40: Chitinase-3-like protein 1 (Chi3-l1)
